# Transmission patterns of *Plasmodium falciparum* by *Anopheles gambiae* in Benin

**DOI:** 10.1186/1475-2875-13-444

**Published:** 2014-11-21

**Authors:** Virgile Gnanguenon, Renaud Govoetchan, Fiacre R Agossa, Razaki Ossè, Frédéric Oke-Agbo, Roseric Azondekon, Arthur Sovi, Roseline Attolou, Kefilath Badirou, Filémon T Tokponnon, Gil G Padonou, Martin C Akogbeto

**Affiliations:** Centre de Recherche Entomologique de Cotonou (CREC), Cotonou, Benin; Faculté des Sciences et Techniques de l’Université d’Abomey-Calavi, Abomey-Calavi, Benin; Université Agricole de Kétou, Kétou, Benin; National Malaria Control Programme, Cotonou, Benin

**Keywords:** Malaria transmission, *Plasmodium falciparum*, *Anopheles gambiae*, Vector control, Benin

## Abstract

**Background:**

To better control malaria, the clear and urgent need is for improved data to inform decision makers, but in several African countries, there is a lack of baseline data on vectors and variation in the intensity of malaria transmission. This has resulted in the implementation of vector control efforts that ignore variation in vector behaviour and intensity of transmission, an approach that is most often not cost-effective. This study presents a detailed entomological description of mosquito distribution and variation in potentially transmissible contacts of *Plasmodium falciparum* following a south to north transect in Benin.

**Method:**

The study was conducted in five locations where environmental parameters were different and malaria prevalence ranged between 14 and 51%. The locations represent the main eco-epidemiological malaria areas in Benin. Mosquitoes were collected using human landing catches, pyrethrum spray catches and windows traps. They were taxonomically and molecularly identified. Head-thoraces of *Anopheles gambiae s.l*. were tested by enzyme-linked immunosorbent assay. Entomological indicators were estimated following WHO guidelines.

**Results:**

The results showed variation between location and period in distribution of *Anopheles coluzzii*, *An. gambiae*, and *Anopheles arabiensis* (p < 0.05). An extension of the reported range of *An. arabiensis* was also observed. Densities of malaria vectors varied significantly between rural and urban sites, however, indoor/outdoor biting ratios remained constant. Proportions of malaria vectors with circumsporozoite protein of *P. falciparum* were similar between locations. The entomological inoculation rates ranged between zero and eight bites/man/night with significant variations between areas.

Four profiles of human exposure to infectious malaria vector bites were observed and included location with one season of high transmission (June - August), two seasons of lower transmission (March-August; October-November), moderate continuous transmission season, and high continuous transmission season of *P. falciparum*.

**Conclusion:**

The study revealed several entomological patterns in transmission of *P. falciparum* in Benin. The data could be used for purposes of planning a more cost-effective vector control strategy, by stratifying the country into higher and lower transmission zones. The information could also be used to guide extension of indoor residual spray based on a targeted use of IRS at sites where the duration of insecticidal effect following spraying coincides with the peak transmission period.

## Background

Malaria vector control strategies have demonstrated their effectiveness through several studies [1–4]. In endemic areas, the result is a significant reduction in malaria morbidity and overall mortality [5–7]. The number of deaths due to malaria decreased from one million in 2000 to 627,000 in 2012 [7]. Several African countries have reported a decline in the number of confirmed malaria cases and the number of malaria-related hospitalizations and deaths. This significant progress in reducing malaria mortality and morbidity was mainly due to the Roll Back Malaria (RBM) initiative, which aimed to reduce at least by 50% the number of malaria deaths in 2010 and 75% by 2015 [8].

However, the development of malaria vector resistance to insecticides is an important threat to the effectiveness of vector control strategies [7]. At the same time, lack of information on vector population dynamics, before and after the application of vector control measures, makes the job of ongoing evaluation and planning of vector control interventions difficult [9].

The priority in terms of malaria control is for improved data to guide policy making [10]. But in most regions of Africa, there is a lack of baseline and monitoring information on vectors and variation in the intensity of malaria transmission, forcing program to use indoor residual spraying (IRS) and long-lasting insecticidal nets (LLINs) in uninformed ways, such as timing IRS such that it misses the peak transmission season. The implementation of these vectors control strategies requires detailed information on malaria transmission and distribution of vector populations. In addition, mosquito density and distribution varies between areas and periods and such variation can change the level of malaria transmission [11]. Assessment of the impact of vector control interventions on malaria transmission requires that study sites be identified and described, with baseline data gathered before the implementation of vector control strategies.

In Benin, current vector control strategies are based on the use of insecticide-based interventions (IRS and LLINs) [1, 12], but the use of these tools could not lead to the elimination of malaria vectors when the deployment of intervention does not account for variability associated with abundance of vectors and intensity of malaria transmission. Existing data on these two indicators are specific, located in a few geographical areas and are not representative of an entire territory.

This study presents a detailed entomological description of the distribution of mosquitoes and the variation in the rate of potentially transmissible contacts of *Plasmodium falciparum* throughout malaria eco-epidemiological areas in Benin. This will help the National Malaria Control Programme to make evidence-based decisions on vector control.

## Methods

### Study design

The study was conducted in five eco-epidemiological locations following a south-to-north transect in Benin where malaria prevalence [13] or geography was different (Table 1). LLIN intervention was ongoing in all selected locations since a universal-coverage national distribution campaign in July 2011. In each location, two representative areas (urban and rural) were selected. The entomological surveys were conducted bimonthly at each area from March to November 2012 during the malaria transmission season, especially when vector density was likely to change due to climatic conditions.

**Table 1:** **Characteristics of each study site**

Location	Geography	Malaria prevalence (%) [13]
**Allada**	Upland, Climate: Coastal-guinean, Cereal area	14.70-26.10
**Dassa**	Upland, Climate: Guinean, Rice area	
**Parakou**	Lowland, Climate: South-Sudanian, Vegetable farms area	26.10-31.10
**Kandi**	Upland, Climate: Sudanian, Cotton area	43.60-51.10
**Malanville**	Lowland, Climate: Sudano-sahelian, Rice area	

Three classical methods were used to monitor vector population dynamics: human landing catches (HLCs) to evaluate man-vector contact, pyrethrum spray collections (PSCs) to assess indoor-resting behavior of vectors, and window exit traps (WETs) to estimate exophilic behaviour. Blood-feeding rates were estimated from mosquitoes collected using PSCs and WETs. Each survey consisted of two-day collections in each of the study sites.

### Study sites

#### Allada

Allada is located in the north of Atlantique Province. The climate is subequatorial with two rainy seasons (March-July and September-November) and two dry seasons (July-September and November-March). The annual average rainfall is between 800 and 1,000 mm. The soil is mainly characterized by earth bar and a marshy depression; it is well suited to food crops of vegetables and fruit and coffee growing. Most lands in Allada are less fertile. The selected areas were:

**Allomey**: an urban area of Allada located 6°40'14.71"N latitude and 2°9'2.99"E longitude.

**Niaouli**: a rural village located 6 °44'37.01"N latitude and 2°8'13.32"E longitude.

#### Dassa

Dassa-Zoume is one of six districts of Collines Province. The climate is Guinean with four seasons: two rainy seasons and two dry seasons. The highest rainfall is recorded between July and September. The vegetation is forests and open savannahs. The two selected areas were:

**Amangassa**: an urban area of Dassa city, located 7°46'31.74"N latitude and 2°11'43.11"E longitude.

**Lema**: a rural village of Dassa, located 7°49'58.83"N latitude and 2°13'37.60"E longitude.

#### Parakou

Parakou is located in the centre of Borgou Province. The climate is South Sudanian. It is characterized by a rainy season (May to October) and a dry season (November-April). The lowest temperatures are recorded between December and January. The average annual rainfall is 1,200 mm. Parakou is watered by tributaries of the Okpara. These rivers remain dry from January to May. The main vegetation is savannah. The selected areas were:

**Zongo zeno**: an urban area of Parakou, located 7°49'58.83"N latitude and 2°13'37.60"E longitude.

**Kpassa**: a rural village of Parakou, located 7°49'58.83"N latitude and 2°13'37.60"E longitude.

#### Kandi

Kandi is located in the centre of Alibori Province in the agro-ecological zone of the cotton area. The climate of Kandi is Sudanese and characterized by two distinct seasons, a rainy season from April to October and a dry season from November to March. December, January and February are characterized by an absolute drought with harmattan. The annual average rainfall varies considerably between 700 and 1,400 mm. The soil is very rugged with predominance of plateau. It is watered by the tributaries of Alibori and Sota. The selected areas were:

**Kossarou**: an urban area of Kandi I, located 11°7'29.32"N latitude and 2°56'9.57"E longitude.

**Sonsoro**: a rural village of Kandi, located 11° 4'58.91"N latitude and 2°13'37.60"E longitude.

#### Malanville

Malanville is located in the far north of the Republic of Benin. The climate is Sudano-Sahelian type and characterized by a dry season from November to April. The average rainfall is 750 mm. Malanville is watered from east to west by the Niger River and its tributaries Alibori, Mekrou and Sota, which are in flood during August and September. The main vegetation is savannah with grassland. The principal agricultural occupation is rice cultivation. The included areas were:

**Arobanda**: an urban area, located 11°51'5.12"N latitude and 3°23'45.06"E longitude.

**Bodjecali**: a rural village, located 11°48'49.06"N latitude and 3°22'58.08"E longitude.

#### Mosquito sampling and field processing

Human Landing Catches (HLCs) were conducted in two houses in each area from 21.00 to 06.00 hours. At each house, mosquito collectors, one outside and one inside, with a flashlight and a mouth aspirator, collected all landing mosquitoes on their feet. Assuming that there is a risk of malaria transmission, collectors received an antimalarial prophylaxis as prevention against malaria. Malaria symptoms were also monitored and treatment was provided when necessary.

A team of two people estimated indoor-resting and outdoor-exiting density using PSCs and WETs in two other houses between 06.45 and 08.00 hours. PSCs consisted of covering all exposed surfaces with white sheets, spraying the rooms and collecting all fallen specimens.

After collection, mosquitoes were counted and morphologically identified [14, 15]. A proportion of unfed *Anopheles gambiae s.l*. females from HLCs were dissected to extract ovaries and to determine parous *An. gambiae s.l*. (mosquitoes that have oviposited at least once) by observing the coiling degree of ovarian tracheoles [16]. Collected females from PSCs and WETs were classified by abdominal status to estimate blood-feeding rate. Collected *An. gambiae s.l*. were stored individually in labelled Eppendorf tubes with desiccant until laboratory processing.

### Laboratory processing

Around 100–150 *An. gambiae s.l*. were randomly selected (from urban and rural areas and proportionally to the number collected in each area) during each collection period for detection of *P. falciparum* circumsporozoite protein (CSP). The heads and thoraces of the *An. gambiae s.l*. females selected were tested by ELISA for identification of *P. falciparum* CSP according to the method described by Wirtz and colleagues [17]. Additionally, for each survey, a random sample of 48 females of *An. gambiae s.l*. by district was identified to species level by molecular method described by Fanello and colleagues [18]. When the number of *An. gambiae s.l*. collected during an assessment did not reach 48 in a district all the *An. gambiae s.l*. collected were used.

### Parameters estimated

Composition of mosquito populations was studied in terms of species diversity, expressed as richness (Taxa S) and species abundance, expressed as evenness [19]. Shannon-Wiener diversity index and Simpson's dominance index was determined at each site.

Human-biting rate (HBR) was defined as the ratio of the total number of mosquito species collected to the total person-nights for the collection period. Endophagy rate represented the proportion of mosquitoes collected indoors against the total of both indoors and outdoors collections from HLC. Exophagy rate was the proportion of mosquitoes collected outdoors against the total of both indoors and outdoors collections from HLC. Endophily was estimated as the percentage of *An. gambiae s.l*. collected by indoor residual (at rest) divided by the total number collected by PSCs and WETs. Exophily rate was determined as the percentage of *An. gambiae s.l*. collected by WETs (exiting) divided by the total number collected by PSCs and WETs. Parity rate was defined as the number of parous *An. gambiae s.l*. divided by the total number dissected. The sporozoite index (SI) was defined as the proportion of total mosquitoes collected found to contain the *P. falciparum* CSP. EIR represented the product of HBR and the SI of night caught mosquitoes.

### Data analysis

Mosquito diversities were compared using Student’s t test. The diversity data were analysed using PAST 2.07. Poisson's confidence intervals method [20] was used to estimate confidence intervals of densities, HBR, EIR, endophagy rates and frequencies of different species of *An. gambiae s.l*. Binomial confidence intervals method [20] was used to estimate confidence intervals of parity rates. Unconditional maximum likelihood estimation method (Wald), or median unbiased estimation (mid-p) of the risk ratio (RR) followed by their confidence intervals obtained using the normal approximation or the exact method mid-p and p-values obtained by Khi2 or midp.exact [21], were used to compare endophagy and exophagy rates and EIR. Principal component analysis was used to reveal the structure of the EIR data to determine the profiles of malaria transmission. Factor 1, 2, and 3 represented principal component 1, 2, and 3.

Zero-inflated regression models [22] were used to predict entomological inoculation rates of unevaluated months due to the important number of zero in the dataset. This model used the EIR as the dependent variable and covariates as: locations and months of collection. This model is the best among the Poisson family models for the data based on the log likelihood. All these parameters were computed and analysed using the free software R version 2.15.1.

### Ethical consideration

This study received ethical approval from the Ministry of Health in Benin. Informed consent was obtained from mosquito collectors who were vaccinated against yellow fever. Mosquito collectors received preventive treatments for malaria and were also subject to regular medical check-ups. Authorization to conduct the study was also obtained from district authorities and from individuals before entering their houses.

## Results

### Mosquito diversity

A total of 8,663 man-biting mosquitoes belonging to five genera (*Anopheles*, *Aedes*, *Culex*, *Mansonia*, *Coquilletidia*) and 21 species were collected in the study sites (Table 2). Two major malaria vectors were found: *An. gambiae s.l*. (found everywhere, and representing 71.1% of the total mosquitoes: 6,166/8,663) and *Anopheles funestus* found in Allada (0.05%) and Dassa (0.1%). *Anopheles nili*, a local vector, was collected but at very low numbers in Parakou (0.04%) and Malanville (0.1%). Other Anophelines collected and identified to species included: *Anopheles pharoensis*, *Anopheles ziemanni*, *Anopheles broheri*, *Anopheles fuscicolor*, and *Anopheles coustani*.

**Table 2:** **Computations for species diversity and dominance index for mosquitoes sampled in sentinel sites**

	Allada		Dassa		Parakou		Kandi		Malanville	
Species	ni	Pi	ni	Pi	Ni	pi	Ni	pi	ni	Pi
*Anopheles gambiae s.l*.	143	0.42	219	0.28	1239	0.59	653	0.69	3912	0.87
*Anopheles funestus*	18	0.05	1	0	0	0	1	0	0	0
*Anopheles nili*	0	0	0	0	1	0	0	0	5	0
*Anopheles pharoensis*	11	0.03	0	0	20	0.01	2	0	71	0.02
*Anopheles ziemanni*	2	0.01	0	0	5	0	0	0	12	0
*Anopheles broheri*	0	0	0	0	0	0	0	0	1	0
*Anopheles coustani*	0	0	0	0	0	0	2	0	1	0
*Anopheles fuscicolor*	0	0	0	0	16	0.01	0	0	0	0
*Aedes aegypti*	6	0.02	0	0	0	0	3	0	10	0
*Aedes vitatus*	6	0.02	0	0	0	0	0	0	40	0.01
*Aedes luteocephalus*	1	0	0	0	2	0	2	0	1	0
*Aedes gr. palpalis*	0	0	0	0	0	0	0	0	25	0.01
*Aedes gr. tarsalis*	0	0	0	0	0	0	1	0	0	0
*Aedes spp*.	0	0	0	0	0	0	13	0.01	4	0
*Culex quinquefasciatus*	43	0.13	546	0.71	455	0.22	250	0.27	293	0.06
*Culex gr decens*	4	0.01	0	0	3	0	0	0	2	0
*Culex nebulosus*	12	0.04	6	0.01	9	0	4	0	10	0
*Culex annulioris*	1	0	0	0	14	0.01	1	0	25	0.01
*Culex fatigans*	3	0.01	0	0	5	0	5	0.01	0	0
*Culex thalassius*	0	0	0	0	0	0	0	0	13	0
*Mansonia africana*	88	0.26	1	0	320	0.15	3	0	0	0
*Coquilletidia cristata*	0	0	1	0	0	0	0	0	97	0.02
**Total**	338	1	774	1	2,089	1	940	1	4,522	1
**Taxas**_**S**	13		6		12		12		17	
**Shannon diversity index**	1.66		0.65		1.12		0.78		0.63	
**Simpson**’**s dominance**	0.27		0.59		0.42		0.56		0.75	

gr = group; ni = number of species i; Pi = fraction of the entire population made up of species i.

*Culex quinquefasciatus* and other *Culex* species were also found at low densities (Table 2). *Culex quinquefasciatus* was the main man-biting mosquitoes collected in Dassa (72%) and the most common non-anbopheline man-biting mosquito species in Parakou (22%) and Kandi (27%), but *Mansonia africana* was the second most common man-biting mosquito species found in Allada (26%) and Parakou (15%). *Coquilletidia cristata* was only found in Malanville and Dassa (Table 2).

Allada recorded Shannon-Wiener diversity and Simpson's dominance index of 1.66 and 0.27, respectively, while Dassa, Parakou, Kandi, and Malanville recorded diversity and dominance values of 0.65 and 0.59, 1.12 and 0.42, 0.78 and 0.56, 0.63 and 0.75, respectively. When the frequencies of diversity and dominance indices of mosquitoes were compared between sites, Allada recorded the highest Shannon-Wiener diversity index and the lowest Simpson's dominance index, while Malanville recorded the highest Simpson's dominance index but the lowest Shannon-Wiener diversity index regardless of the number of collected species in this area was high. In Allada, many species were collected but at very low densities. There was no significant difference in mosquito diversity between Allada, Parakou, Kandi, and Malanville (p > 0.05), but in the district of Dassa a significant difference (p < 0.001) in mosquito diversity was observed compared to other districts.

### Distribution of *Anopheles gambiae*species complex

**Figure 1:**
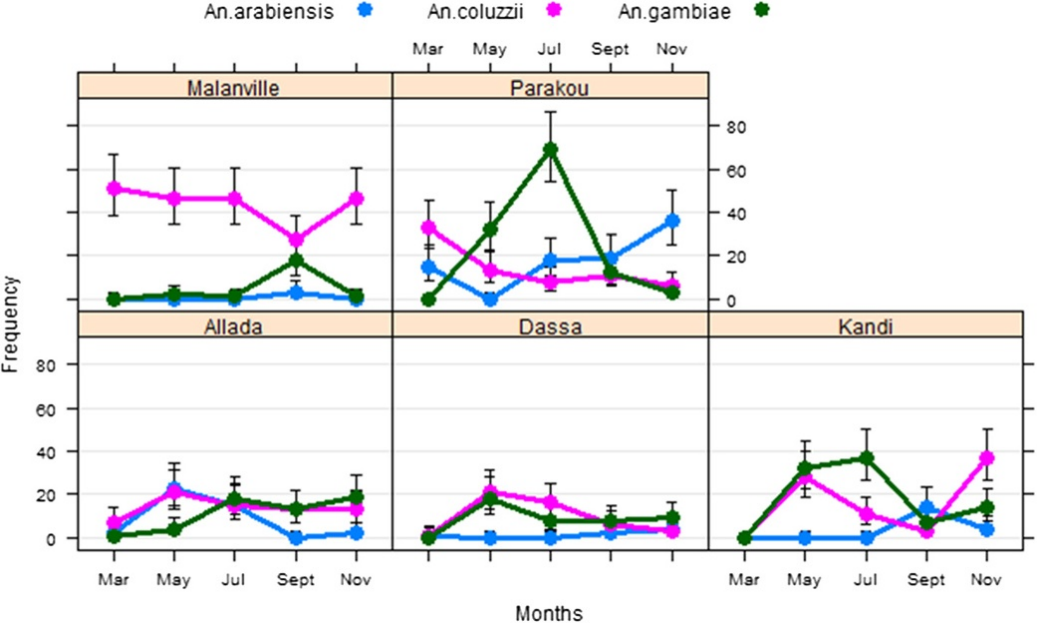
**Distribution of species of the complex** ***Anopheles gambiae*** **.**

In total, 966 *An. gambiae s.l*. were analysed by PCR for species identification. Three species of the complex *An. gambiae* were identified: *Anopheles coluzzii*, *An. gambiae* and *Anopheles arabiensis. Anopheles coluzzii* represented 50% (n = 482) of mosquitoes tested against 16% (n = 158) of *An. arabiensis*, and 34% (n = 326) of *An. gambiae*. The three species were found at five sites but at different times during the transmission season (Figure 1). *Anopheles arabiensis* previously limited in Parakou was identified in Dassa and Allada. *Anopheles arabiensis* (43%) and *An. coluzzii* (48%) predominated between March and May at Allada. From July to November, density of *An. arabiensis* decreased to 16% while density of *An. gambiae* increased from 9 to 46%. From March to July, *An. coluzzii* and *An. gambiae* were the most represented species in Dassa (58 and 40%, respectively) and Kandi (36 and 64%). *Anopheles arabiensis* were found in a sizeable proportion (19-23%) at these two areas between September and November (Figure 1). *Anopheles coluzzii* was highly predominant (p < 0.05) in Malanville. In Parakou, *An. gambiae* predominated between May and July, while *An. arabiensis* was mostly found between September and November, and *An. coluzzii* in March (Figure 1).

### Aggressive density of *Anopheles gambiae s.l*

**Figure 2:**
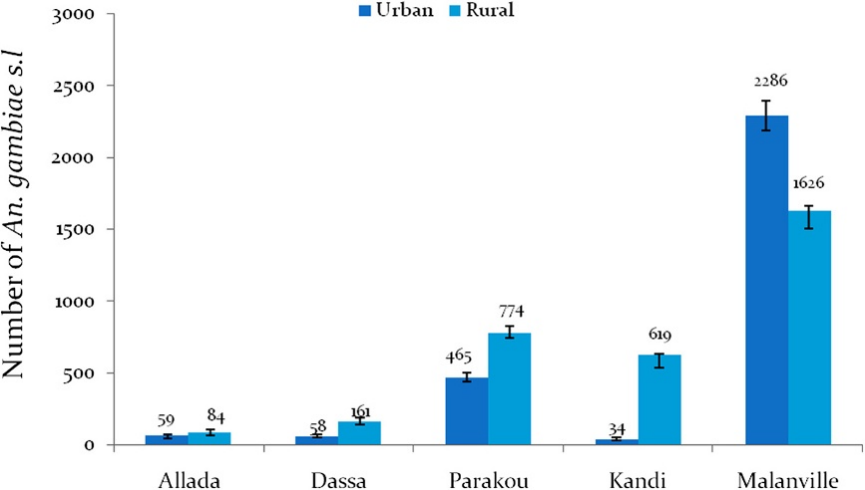
**Aggressive densities of** ***Anopheles gambiae s.l.*** **in urban and rural areas.**

A total of 143 (117.74-165.34) *An. gambiae s.l*. were collected in Allada, 219 (194.59-246.39) in Dassa, and 1,239 (1,184.32-1,296.88) in Parakou. Respectively, 653 and 3,912 *An. gambiae s.l*. were collected in Kandi and Malanville. The number of *An. gambiae s.l*. was generally higher in rural areas than in urban areas except in Malanville where the opposite was observed (Figure 2).

**Figure 3:**
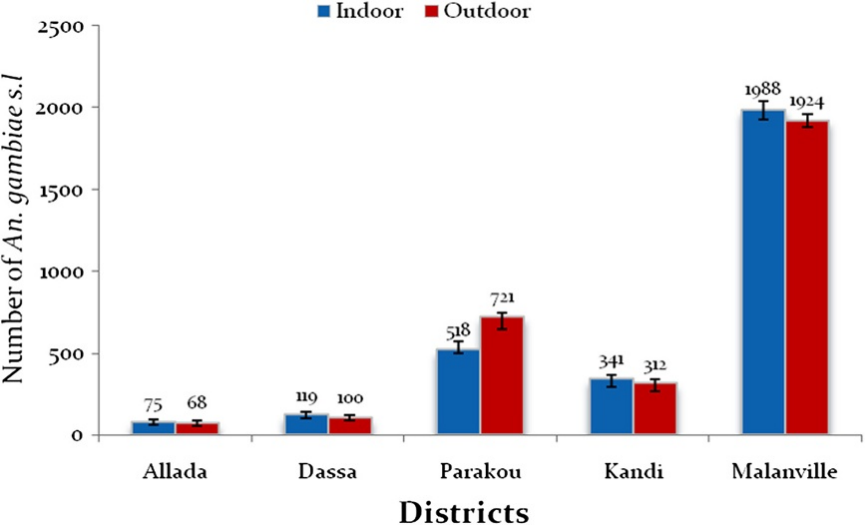
**Aggressive densities of** ***Anopheles gambiae s.l.*** **indoors and outdoors.**

In Malanville the average HBR was 49 bites/man/night. The aggressive density of *An. gambiae s.l*. observed in Malanville was significantly higher than in Parakou (15 bites/man/night), Kandi (eight bites/man/night), Dassa (three bites/man/night), and Allada (two bites/man/night). Endophagy rates and exophagy rate were similar in Malanville, Kandi Dassa, and Allada (p > 0.05). Exophagy rate of *An. gambiae s.l*. was significantly higher than the endophagy rate (p < 0.001) at Parakou (Figure 3).

### Aggressive density of other Culicidae

**Figure 4:**
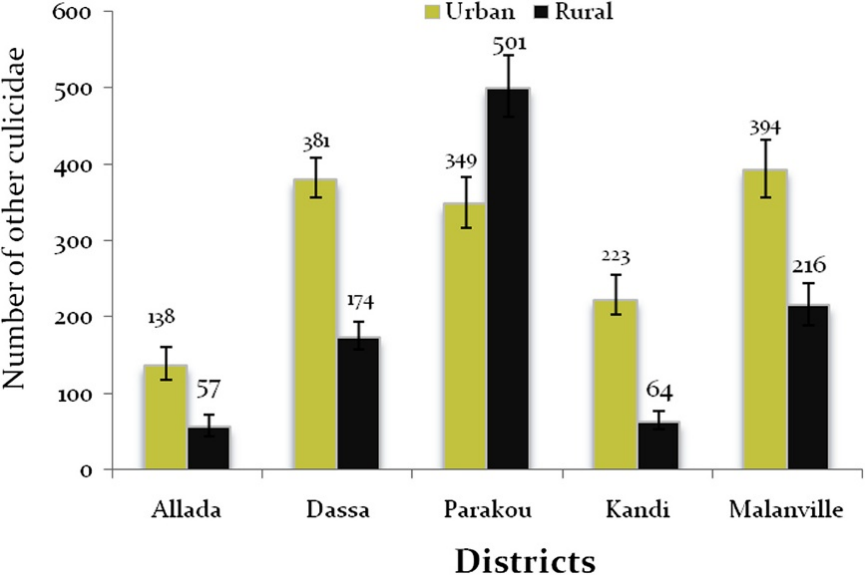
**Aggressive densities of other mosquito species.**

**Figure 5:**
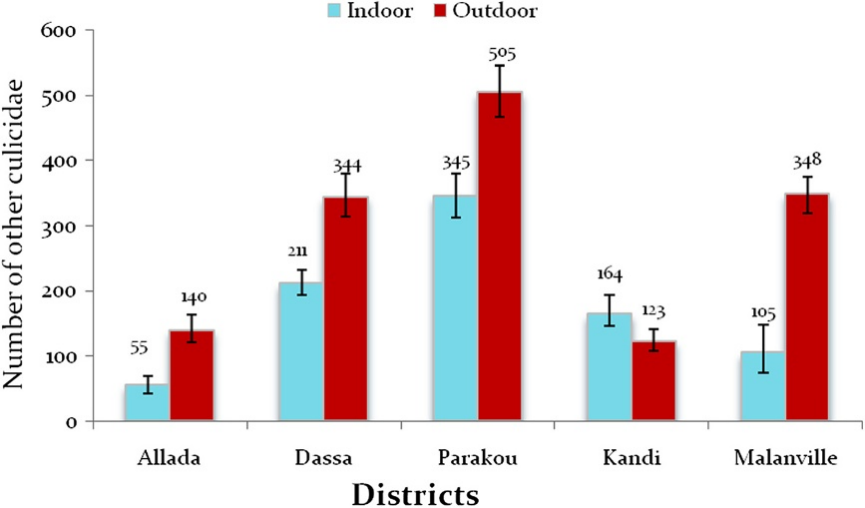
**Aggressive densities of other Culicidae indoors and outdoors per district.**

The abundance of other mosquito species was significantly lower compared to that of *An. gambiae s.l*. (p < 0.05) but also varied from one site to another. Unlike *An. gambiae s.l*., other Culicidae were more abundant in urban than in peripheral environment in all districts except Parakou where other Culicidae were significantly higher (501 (462.50-542.303)) in peripheral areas (349 (317.19-383.87)) (Figure 4). The number of other Culicidae ranged from 138 (118.31-160.66) to 394 (356.34-432.57) in urban areas in Allada, Dassa, Kandi, and Malanville. In rural areas of these districts, the number of other Culicidae was between 57 (44.80-72.25) and 216 (189.13-244.85). Fifty-five (41.94-69.09) to 345 (313.26-379.78) human-biting other Culicidae were collected indoors (Figure 5). The number of other mosquito species collected outdoors varied from 123 (108.78-140.80) to 348 (318.65-375.24) (Figure 5). The endophagy rates of other Culicidae were significantly lower than exophagy rates in all districts (p < 0.05) except in Kandi where endophagy rate was significantly higher than exophagy rate (p < 0.05).

### Longevity of *Anopheles gambiae s.l*

Some 1,810 ovaries of *An. gambiae s.l*. were dissected. The majority of *An. gambiae s.l*. dissected were old with an average parity rate of 88.06%. The parity rate was 88.11% in Allada and 76.87% in Dassa (Table 3). In Parakou, Kandi and Malanville, the observed parity rates were 88.60, 90.32 and 88.34%, respectively. No significant variation was observed between sites (p > 0.05).

**Table 3:** **Parity rates of** ***Anopheles gambiae s.l***.

Localities	Total	N parous	Parity rate (%)	P
Allada	143	126	88.11	0.2878
Dassa	134	103	76.87	
Parakou	579	513	88.60	
Kandi	465	420	90.32	
Malanville	489	432	88.34	
**Total**	1,810	1,594	88.06	

N = Number; P = P-value.

### Exophily of *Anopheles gambiae s.l*., *Mansonia spp*. and *Culex spp*

A total of 2,677 mosquitoes were collected in morning PSCs and WETs (Table 4). They belonged to three genera (*Anopheles* including *An. gambiae s.l*. only, *Mansonia* and *Culex*). 1,167 *An. gambiae s.l*. were collected *versus* 1,510 *Mansonia* and *Culex spp* (Table 4). Observed exophily of *An. gambiae s.l*. was generally low (Table 4). Exophily rates for *An. gambiae s.l*. in Allada, Parakou, and Kandi were similar (p > 0.05) with respective exophily rates of 17.24, 17.65, and 21.34 (Table 4). The highest exophily rate was observed in Dassa (52.17%, p = 0.03). The lowest exophily rate was observed in Malanville (2.95%, p < 0.0001). Observed exophily rates in *Mansonia* and *Culex spp* in Parakou (30.65%) and Malanville (1.41%) was significantly lower than that observed in other districts (p < 0.05). Exophily rates in Allada, Dassa and Kandi were 57.58, 39.60, and 39.20%, respectively.

**Table 4:** **Observed exophily rates of mosquitoes collected by study site**

Area	***Anopheles gambiae s.l.***				***Mansonia***and ***Culex spp***			
	SPCs	WETs	Exophily (%)	P	SPC	WETs	Exophily (%)	P
Allada	24	5	17.24	-	14	19	57.58	-
Dassa	11	12	52.17	0.03	122	80	39.60	0.14
Parakou	42	9	17.65	0.96	86	38	30.65	0.02
Kandi	199	54	21.34	0.33	183	118	39.20	0.17
Malanville	787	24	2.95	<0.0001	838	12	1.41	<0.0001
**Total**	**1,** **063**	**104**	**8.91**		**1,** **243**	**267**	**17.67**	

PSCs = Pyrethrum Spray Collections; WETs = Window Exit Traps.

### Mosquito blood-feeding rates

The observed blood-feeding rates of mosquitoes collected from indoor residual and WETs in different study sites were high (Table 5). *Anopheles gambiae s.l*. had a higher blood-feeding rate in Allada (100%) and Malanville (93.65%) than in Dassa (56.52%) and Kandi (73.87%). In Parakou, *An. gambiae s.l*. had a similar blood-feeding rate (80.39%) to all other districts (Table 5). A significant variation in blood-feeding behaviour of *An. gambiae s.l*. was observed between sites (p < 0.05).

**Table 5:** **Blood**-**feeding rates of** ***Anopheles gambiae s.l***., ***Mansonia*** **and** ***Culex spp***

	***Anopheles gambiae s.l.***				***Mansonia***and ***Culex spp***			
Areas	Total	Fed	Blood-feeding rate (%)	P	Total	Fed	Blood-feeding rate (%)	P
Allada	29	29	100.0^a^	0.0004	33	29	87.87^a^	0.0009
Dassa	23	13	56.52^b^		202	97	48.01^b^	
Parakou	51	41	80.39^ab^		124	90	72.58^a^	
Kandi	253	187	73.91^b^		301	230	76.41^a^	
Malanville	811	759	93.58^a^		850	790	92.94^a^	

Blood-feeding rates which carry same letters in exposant were not significantly different (p > 0.05); P = P-value.

*Mansonia spp* and *Culex spp* also had high blood-feeding rates (Table 5). The observed blood-feeding rates among these species varied between sites (p < 0.05) and ranged from 48 to 93%.

### Infectivity of *Anopheles gambiae s.l*

A total of 1,652 head-thoraces of *An. gambiae s.l*. collected were assessed with CSP-ELISA assays. The mean SI was 4.46%. SI was similar (p > 0.05) in urban (4.3%: 24 head-thoraces+/556 head-thoraces) and rural areas (4.5%: 50 head-thoraces +/1,102 head-thoraces). The average SI was 5.8% (31 head-thoraces +/531 head-thoraces) in Parakou, 5.6% (12 head-thoraces +/214 head-thoraces) in Dassa, 2.5% (seven head-thoraces +/280 head-thoraces) in Kandi, 4.4% (eight head-thoraces +/182 head-thoraces) in Allada, and 3.5% (16 head-thoraces+/451 head-thoraces) in Malanville. The highest infection rate was observed in urban Dassa, and the lowest was observed in rural Kandi (Table 6). In urban Kandi, no positive head-thorax was found. *Plasmodium falciparum* infection was observed in *An. gambiae s.l*. from March to November in urban Malanville and rural Parakou. In the other locations *P. falciparum* infection rate was found variable over time.

**Table 6:** ***Plasmodium falciparum*** **infection rates observed in** ***Anopheles gambiae s.l***.

Localities		March	May	July	September	November	SI/Period	RR	95% CI	P
**Allada urban**	**Thorax**	4	33	23	13	14	5.75	1.00	_	0.49
	**Thorax**+	0	2	1	1	1				
	**SI**	0	6.06	4.35	7.69	7.14				
**Allada rural**	**Thorax**	7	24	24	20	16	3.30	0.97	0.91-1.04	
	**Thorax**+	0	1	0	0	2				
	**SI**	0	4.17	0	0	12.5				
**Dassa urban**	**Thorax**	0	18	23	5	7	6.86	1.00	_	0.73
	**Thorax**+	0	0	0	1	1				
	**SI**	0	0	0	20	14.29				
**Dassa rural**	**Thorax**	2	60	77	13	9	5.38	1.03	0.96-1.10	
	**Thorax**+	0	3	5	2	0				
	**SI**	0	5	6.49	15.38	0				
**Parakou urban**	**Thorax**	19	40	44	29	50	5.39	1.00	_	0.57
	**Thorax**+	1	0	5	3	0				
	**SI**	5.26	0	11.36	10.34	0				
**Parakou rural**	**Thorax**	125	50	46	78	50	4.71	1.01	0.97-1.06	
	**Thorax**+	16	3	1	2	0				
	**SI**	12.8	6	2.17	2.56	0				
**Kandi urban**	**Thorax**	0	9	3	24	4	0	1.00	_	0.60
	**Thorax**+	0	0	0	0	0				
	**SI**	0	0	0	0	0				
**Kandi rural**	**Thorax**	0	87	90	60	1	1.8	-	_	
	**Thorax**+	0	3	2	2	0				
	**SI**	0	3.45	2.22	3.33	0				
**Malanville urban**	**Thorax**	11	41	48	40	50	4.84	1.00	_	0.61
	**Thorax**+	1	1	2	1	3				
	**SI**	9.09	2.44	4.17	2.5	6				
**Malanville rural**	**Thorax**	67	59	47	38	50	2.72	0.99	0.95-1.03	
	**Thorax**+	4	2	2	0	0				
	**SI**	5.97	3.39	4.26	0	0				

SI = Sporozoïte Index; RR = Rate Ratio; CI = Confidence Interval; P = P-value.

### Entomological inoculation rate of *Anopheles gambiae s.l*

Table 7 shows the distribution of EIR by site. The EIR of *An. gambiae s.l*. was high in Malanville, with an average range of four to eight infected bites/man/night during the study period. Infected bites were recorded each month from March to November at this location. Parakou was also characterized by a high malaria transmission with two to five infected bites/man/night in rural and urban areas. The lowest EIR was observed in urban Kandi with no infected bites/man/night. Globally, observed EIRs were significantly higher in rural areas than in urban areas (p < 0.05), except in Allada where EIRs were similar (Table 7). EIR was generally higher in July than in other months (Table 7).

**Table 7:** **Observed entomological inoculation rates** (**EIR**) **by study site**

Localities	March	May	July	September	November	EIR/Night	EIR/Month	EIR/Year	RR/IC95	P
**Allada urban**	4.00	22.00	18.00	10.00	14.00	0.48	14.37	174.82	1.00	0.45
**HBR/** **night**	0.50	2.75	2.25	1.25	1.75					
**EIR/** **night**	0.00	0.16	0.10	0.10	0.12					
**Allada rural**	7.00	10.00	23.00	19.00	16.00	0.30	9.06	110.27	0.50 (0.09-2.73)	
**HBR/** **night**	0.88	1.25	2.87	2.38	2.00					
**EIR/** **night**	0.00	0.05	0.00	0.00	0.25					
**Dassa urban**	0.00	17.00	21.00	5.00	7.00	0.25	7.50	91.26	1.00	0.022
**HBR/** **night**	0.00	2.12	2.62	0.63	0.88					
**EIR/** **night**	0.00	0.00	0.00	0.13	0.13					
**Dassa rural**	2.00	50.00	103.00	9.00	5.00	1.32	39.62	482.09	5.00 (1.10-22.81)	
**HBR/** **night**	0.25	6.25	12.87	1.13	0.63					
**EIR/** **night**	0.00	0.31	0.84	0.17	0.00					
**Parakou urban**	19.00	278.00	88.00	29.00	51.00	1.75	52.48	638.51	1.00	0.00
**HBR/** **night**	2.38	34.75	11.00	3.63	6.38					
**EIR/** **night**	0.12	0.00	1.25	0.37	0.00					
**Parakou rural**	125.00	276.00	161.00	90.00	122.00	4.79	143.84	1,750.03	2.13 (1.30-3.50)	
**HBR/** **night**	15.63	34.50	20.12	11.25	15.25					
**EIR/** **night**	2.00	2.07	0.44	0.29	0.00					
**Kandi urban**	0.00	4.00	3.00	23.00	4.00	0	0	0	1.00	<0.0001
**HBR/** **night**	0.00	0.50	0.38	2.88	0.50					
**EIR/** **night**	0.00	0.00	0.00	0.00	0.00					
**Kandi rural**	0.00	142.00	372.00	104.00	1.00	2.08	62.33	758.31	-	
**HBR/** **night**	0.00	17.75	46.50	13.00	0.13					
**EIR/** **night**	0.00	0.61	1.03	0.43	0.00					
**Malanville urban**	11.00	120.00	1042.00	522.00	591.00	8.44	253.20	3,080.64	1.00	0.0012
**HBR/** **night**	1.38	15.00	130.25	65.25	73.87					
**EIR/** **night**	0.12	0.37	5.43	1.63	4.43					
**Malanville rural**	67.00	305.00	515.00	159.00	580.00	4.53	136.04	1,655.14	0.52 (0.37-0.73)	
**HBR/** **night**	8.38	38.12	64.38	19.87	72.50					
**EIR/** **night**	0.50	1.29	2.74	0.00	0.00					

EIR = Entomological Inoculation Rate; RR = Rate Ratio; CI = Confidence Interval; P = P-value.

Principal component analysis identified three significant factors that accounted for around 90% of the variation in EIR (Table 8). Observed human exposure to infectious bites in Kandi, Dassa and Allada significantly contributed to the formation of factor 1, transmission risk in Parakou essentially contributed to the formation of factor 2, and those of Malanville were decisive in factor 3 (Table 8).

**Table 8:** **Contribution of entomological inoculation rate observed in each location to factors 1**-**3**

Factors	Localities	Correlation	P
Factor 1	Kandi	0.9330095	0.0000812
	Dassa	0.8986404	0.0004079
	Allada	-0.703905	0.0230912
Factor 2	Parakou	-0.816829	0.0039228
Factor 3	Malanville	0.9605642	0.0000101

**Figure 6:**
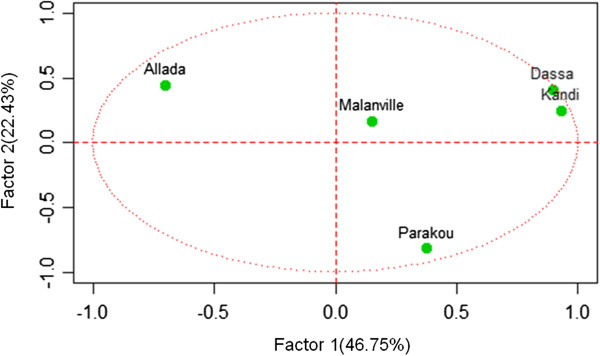
**Representation of entomological inoculation rate of study sites on axes 1 and 2.**

**Figure 7:**
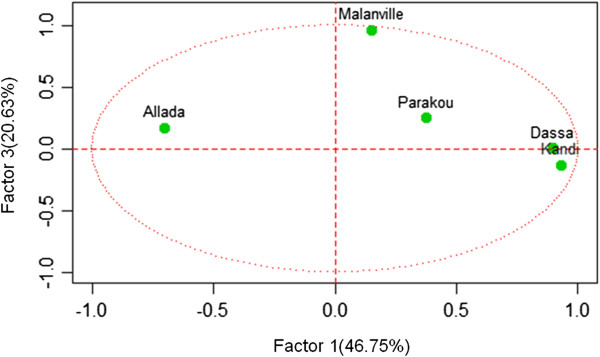
**Representation of entomological inoculation rate of study sites on axes 1 and 3.**

**Figure 8:**
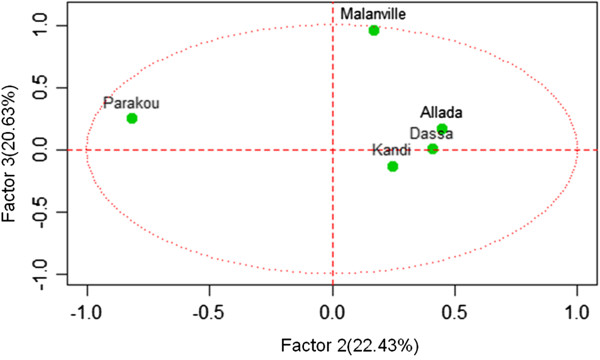
**Representation of entomological inoculation rate of study sites on axes 2 and 3.**

**Figure 9:**
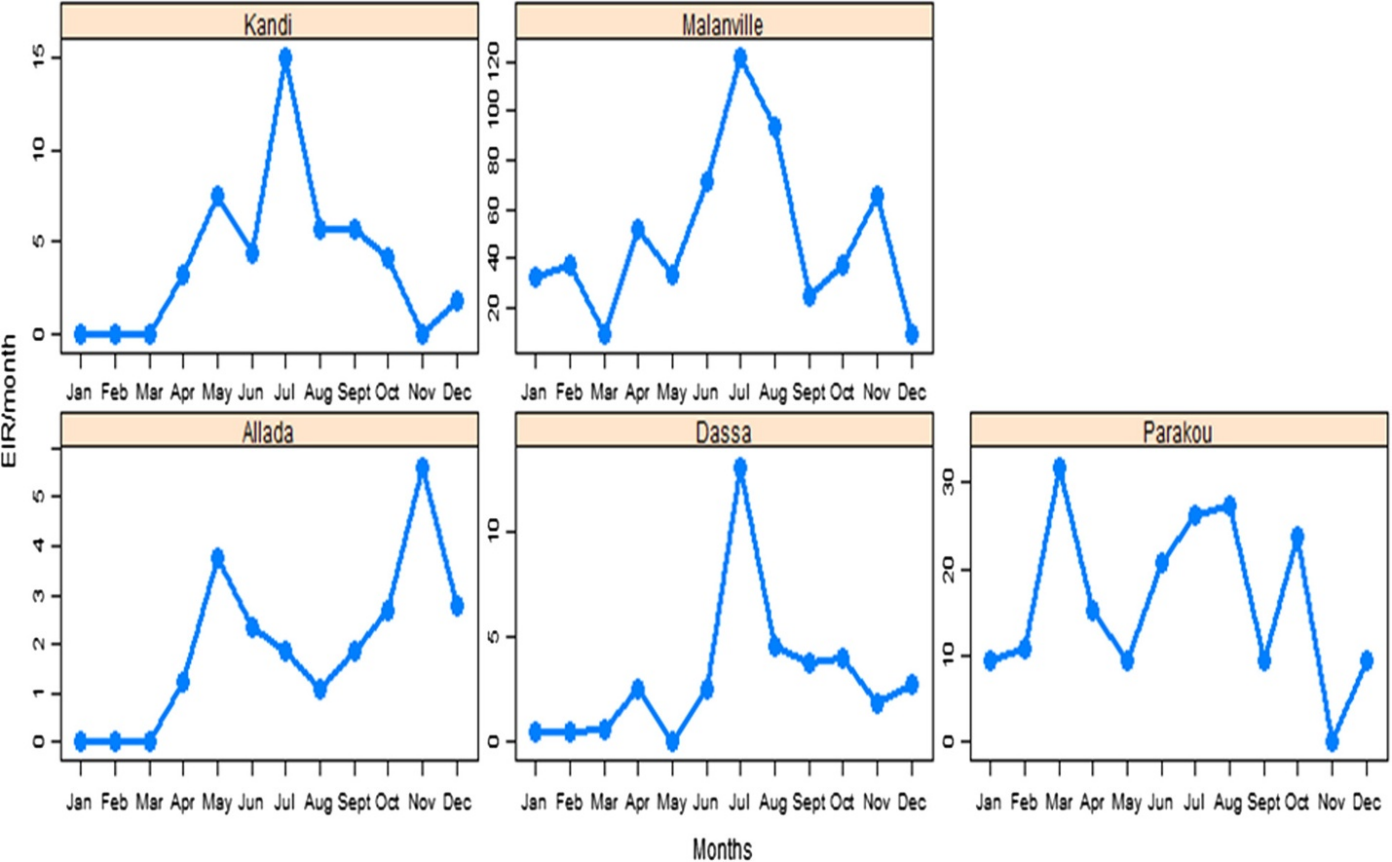
**Predicted entomological inoculation rate per month in each study site with different scales of axes.**

Figures 6, 7 and 8 describe the relationship between observed EIRs and these factors. The results suggest four profiles of malaria transmission. The first profile includes Kandi and Dassa where variations in EIRs were similar (Figure 6). In contrast, Allada had a different profile suggesting that variation in entomological inoculation rates was significantly different from those observed in Dassa and Kandi (Figure 7). Variations in EIRs in Malanville and Parakou were significantly different, suggesting two different malaria transmission profiles (Figure 8). The existence of such profiles was further confirmed by a monthly EIR prediction model in each site (Figure 9). The predicted EIR model showed four trends (profiles) in malaria transmission. The first trend included transmission predicted in Dassa and Kandi that showed one season of high transmission. This season begins in June and ends in August with a maximum of 13 infectious bites/man/month in Dassa *versus* 15 infectious bites in Kandi (Figure 9). The second trend was determined by the transmission predicted in Allada where transmission is lower than in other localities but with two seasons. The first season begins in March and ends in August with a maximum of four infectious bites/man/month. The second transmission season occurs in October and ends in November with a maximum of six infectious bites/man/month. The third transmission trend was determined by EIR predicted in Parakou with three peaks suggesting a continuous malaria transmission in this area. In this area, EIRs varied from nine to 27 infectious bites/man/month (Figure 9). The fourth trend observed in Malanville showed several peaks of EIR ranging from nine to 120 infectious bites/man/month (Figure 9). It also suggests a continuous transmission in this area but with higher value than in Parakou.

## Discussion

The majority, seventy one percent, of the vectors collected during this study were *An.gambiae s.l*. Abundance varied according to the degree of urbanization. Estimates of indoors versus outdoors biting were similar. The number of *An. gambiae s.l*. infective bites/human/night varied between locations. Based on these results four *P. falciparum* transmission profiles, across a North–south transect in Benin, are described.

No significant variation in vector bio-diversity was observed between locations, suggesting that similar control intervention would work in each location, thereby reduce higher costs associated with the application of different control methods in different locations [23]. Taxonomic results also demonstrate a spatio- temporal variation in the complex *An. gambiae* as observed in Nigeria, Cameroon and Burkina Faso [24–26].

*Anopheles arabiensis*, a vector of dry savannah areas and limited in 1980 between the extreme north Benin (Malanville) and Parakou [27, 28], was found in Dassa and Allada in 2012. The results suggest an extension of the spatial distribution of this vector associated with destruction of forests and large areas (70,000 ha/yr) for agriculture, hunting, grazing, and wood harvesting in central and southern Benin [29]. Habitat changes, such as these, may contribute to the spread of this malaria vector in southern Benin. In addition, long dry seasons recently observed in Benin , and elsewhere in Africa, due to climate change [30], foster the spread of *An. arabiensis* that has a similar abudance with *An. gambiae* during certain months (May, September and November).

Differences between densities of *An. gambiae s.l*. and other man-biting mosquitoes in each site could be explained by the ecological characteristics of the environment. Many of the study sites, such as Malanville and Parakou, are suitable for vector breeding. They are lowland areas where rice and cotton cultivation, and vegetable farming are the main activities [31, 32]. Densities of *An. gambiae s.l*. were similar inside and outside houses. This result was contrary to that observed by Akogbeto and colleagues [1] showing a low endophagy rate in indoor residual spray areas. It highlighted the importance of such strategies on malaria vector control. High densities observed in urban Malanville are due to the existence of rice-growing areas with permanent mosquito breeding sites in the urban area [31].

The EIR estimates human exposure to malaria-infected vectors. It is commonly used to assess malaria endemicity and transmission intensity [33]. In the present study, observed EIRs varied significantly between locations. The highest rates were observed in Parakou and Malanville with more than two infectious bites/man/night suggesting that populations from Malanville and Parakou are more exposed to transmission of *P. falciparum* compared to other locations. Observed malaria transmission risk at each location could be associated with their environmental characteristics. Koudou and colleagues [34] reported an increase EIR from 38 to 295 infective bites/person/year with a return to rice cultivation at Zitta region in Côte d’Ivoire. Permanent exposure to malaria for a community close to vegetable farming was also reported by Yadouleton *et al*. [35]. Such observations suggest that rice cultivation and vegetable farming are key factors associated with high malaria transmission risk and could explain transmission observed in Malanville and Parakou. Rice cultivation could also explain differences in EIR between Dassa and Allada, which are both highlands with similar malaria prevalence, and between Kandi and Malanville which have similar malaria prevalence. In addition, Kandi, Diassa, and Allada are highlands reported to have lower malaria transmission risk than the lowlands (Malanville and Parakou) [36]. The impact of the current LLINs intervention on vector populations may also explain the lower vector densities and lower transmission risks than expected in some areas where case data-based malaria prevalence does not seem to correlate with EIRs [3, 37]. But a firm conclusion on correlation between EIRs and prevalence will be drawn if entomological and parasitological data are concurrently collected.

Infectious-vectors bites were higher in rural areas compared to urban areas in a same district suggesting a spatial heterogeneity of EIRs. This shows the importance of local conditions or micro-ecological conditions and environment characteristics in the intensity of malaria transmission.

High EIRs observed in certain locations was particularly due to a high HBR of *An. gambiae*, but not to a high infectious rate of vectors. This observation raises the question: where are we most vulnerable to malaria: between areas with low densities of vectors with high infectious rates or areas with high densities of vectors with low infectious rates?

Observed patterns in transmission of *P. falciparum* included locations with one season of high malaria transmission, locations with two seasons of high transmission and locations with continuous high transmission of malaria. Malanville and Parakou are locations at high risk of transmission where urgent, efficient vector control strategies should be implemented to help populations.

The study demonstrates the role of vector biology in the epidemiology of malaria in Benin. However, the lack of data on malaria prevalence estimated by microscopy or rapid detection in populations of each study site represents an important limitation. The study could also be improved by increasing the number of surveys and including more locations.

The study of intensity of *P. falciparum* transmission by mosquitoes is very important for implementation of insecticide-based vector control interventions in Benin where several large-scale vector control interventions (LLINs and IRS) are ongoing. It will guide policies in the selection of priority areas for vector control interventions. Knowledge of the number of high transmission season in each area will also help to implement cost-effective IRS interventions.

## Conclusion

This study documents at least four vector-biology-associated patterns in transmission of *P. falciparum* from southern to northern Benin. It shows a spatiotemporal variation in the distribution of malaria vectors. It also reveals a spatial heterogeneity in human-biting behaviour as well as variation in human exposure to infectious bites of malaria vectors.

These data represent a source of information that could guide implementation of any cost-effective vector control strategy in Benin by targeting the higher transmission areas, that is by stratifying the country into higher and lower transmission zones. The study could also help extension of indoor residual spray based on a targeted use of IRS at sites where the duration of insecticidal effect following spraying coincides with the peak transmission period.

## References

[1] Akogbeto M, Padonou GG, Bankole HS, Gazard DK, Gbedjissi GL (2011). Dramatic decrease in malaria transmission after large-scale indoor residual spraying with bendiocarb in Benin, an area of high resistance of *Anopheles gambiae* to pyrethroids. Am J Trop Med Hyg.

[2] West PA, Protopopoff N, Rowland MW, Kirby MJ, Oxborough RM, Mosha FW, Malima R, Kleinschmidt I (2012). Evaluation of a national universal coverage campaign of long-lasting insecticidal nets in a rural district in north-west Tanzania. Malar J.

[3] Sovi A, Azondékon R, Aïkpon RY, Govoétchan R, Tokponnon F, Agossa F, Salako AS, Oké-Agbo F, Aholoukpè B, Okè M, Gbénou D, Massougbodji A, Akogbéto M (2013). Impact of operational effectiveness of long-lasting insecticidal nets (LLINs) on malaria transmission in pyrethroid-resistant areas. Parasit Vectors.

[4] Tokponnon FT, Aholoukpe B, Denon EY, Gnanguenon V, Bokossa A, N’guessan R, Oke M, Gazard DK, Akogbeto MC (2013). Evaluation of the coverage and effective use rate of long-lasting insecticidal nets after nation-wide scale up of their distribution in Benin. Parasit Vectors.

[5] Lengeler C (2004). Insecticide-treated bed nets and curtains for preventing malaria. Cochrane Database Syst Rev.

[6] WHO (2012). World Malaria Report 2012.

[7] WHO (2013). World Malaria Report 2013.

[8] WHO (2010). WHO / World Malaria Report 2010.

[9] Killeen GF, Okumu FO, N’Guessan R, Coosemans M, Adeogun A, Awolola S, Etang J, Dabiré RK, Corbel V (2011). The importance of considering community-level effects when selecting insecticidal malaria vector products. Parasit Vectors.

[10] Marsh K (2010). Research priorities for malaria elimination. Lancet.

[11] Munga S, Yakob L, Mushinzimana E, Zhou G, Ouna T, Minakawa N, Githeko A, Yan G (2009). Land use and land cover changes and spatiotemporal dynamics of Anopheline larval habitats during a four-year period in a highland community of Africa. Am J Trop Med Hyg.

[12] Gnanguenon V, Azondekon R, Oke-Agbo F, Beach R, Akogbeto MC (2014). Durability assessment results suggest a serviceable life of two, rather than three, years for the current long-lasting insecticidal (mosquito) net (LLIN) intervention in Benin. BMC Infect Dis.

[13] MDAEP, INSAE, Bénin ICF International (2013). Enquête Démographique et de Santé (EDSB-IV) 2011–2012.

[14] Gillies MT, De Meillon B (1968). The Anophelinae of Africa South of the Sahara.

[15] Gillies MT, Coetzee M (1987). A supplement to the anophelinae of Africa south of the Sahara. Publ South Afri Inst Med Res.

[16] Detinova T, Gillies M (1964). Observations on the determination of the age composition and epidemiological importance of populations of *Anopheles gambia*e Giles and *Anopheles funestus* Giles in Tanganyika. Bull World Health Organ.

[17] Wirtz R, Ballou W, Schneider I, Chedid L, Gross M, Young J, Hollingdale M, Diggs C, Hockmeyer W (1987). *Plasmodium falciparum*: immunogenicity of circumsporozoite protein constructs produced in *Escherichia coli*. Exp Parasitol.

[18] Fanello C, Della TA (2001). Distribution du gène Kdr dans les formes chromosomiques et moléculaires des Anopheles gambiae s.s en Afrique Occidentale.

[19] Simpson EH (1949). Measurement of diversity. Nature.

[20] Kenneth R (2012). Epidemiology An Introduction.

[21] Nicolas PJ (2003). Statistics for Epidemiology.

[22] Lambert P-W (1992). Zero-inflated Poisson regression, with an application to defects in Manufacturing. Technometrics.

[23] Lingenfelser A, Rydzanicz K, Kaiser A, Becker N (2010). Mosquito fauna and perspectives for integrated control of urban vector-mosquito populations in southern Benin (West Africa). Ann Agric Environ Med.

[24] Onyabe Y, Conn E (2003). Population genetic structure of the malaria mosquito *Anopheles arabiensis* across Nigeria suggests range expansion. Mol Ecol.

[25] Wondji C, Simard M, Fontenille D (2002). Evidence for genetic differentiation between the molecular forms M and S within the Forest chromosomal form of *Anopheles gambiae* in an area of sympatry. Insect Mol Biol.

[26] Dabiré R, Diabaté A, Namountougou M, Toé K, Ouari A, Kengne P, Bass C, Baldet T (2009). Distribution of pyrethroid and DDT resistance and the L1014F kdr mutation in *Anopheles gambiae s.l*. from Burkina Faso (West Africa). Trans R Soc Trop Med Hyg.

[27] Akogbeto M (1992). Etude des aspects epidemiologiques du paludisme côtier lagunaire au Bénin. PhD thesis.

[28] Akogbeto M (1995). Entomological study on the malaria transmission in coastal and lagoon areas: the case of a village built on a brackish lake. Ann Société Belge Médecine Trop.

[29] FAO (2002). The State of Food Insecurity in the World.

[30] Baudoin M-A, Sanchez AC, Fandohan B (2013). Small scale farmers’ vulnerability to climatic changes in southern Benin: the importance of farmers’ perceptions of existing institutions. Mitig Adapt Strateg Glob Change.

[31] Yadouleton A, Asidi A, Djouaka R, Braïma J, Agossou C, Akogbeto M (2009). Development of vegetable farming: a cause of the emergence of insecticide resistance in populations of *Anopheles gambiae* in urban areas of Benin. Malar J.

[32] Yadouleton A, Martin T, Padonou G, Chandre F, Asidi A, Djogbenou L, Dabiré R, Aïkpon R, Boko M, Glitho I, Akogbeto M (2011). Cotton pest management practices and the selection of pyrethroid resistance in *Anopheles gambiae* population in Northern Benin. Parasit Vectors.

[33] Fontenille D, Cohuet A, Awono-Ambene P, Kengne P, Antonio-Nkondjio C, Wondji C, Simard F (2005). Vecteurs de paludisme: du terrain à la génétique moléculaire. Rev Epidémiologie Santé Publique.

[34] Koudou BG, Adja AM, Matthys B, Doumbia M, Cisse G, Kone M, Tanner M, Utzinger J (2007). Pratiques agricoles et transmission du paludisme dans deux zones éco-épidémiologiques au centre de la Côte d’Ivoire. Bull Société Pathol Exot.

[35] Yadouléton A, N’guessan R, Allagbé H, Asidi A, Boko M, Osse R, Padonou G, Kindé G, Akogbéto M (2010). The impact of the expansion of urban vegetable farming on malaria transmission in major cities of Benin. Parasit Vectors.

[36] Omukunda E, Githeko A, Ndong’a MF, Mushinzimana E, Atieli H, Wamae P (2013). Malaria vector population dynamics in highland and lowland regions of western Kenya. J Vector Borne Dis.

[37] Terlouw DJ, Morgah K, Wolkon A, Dare A, Dorkenoo A, Eliades MJ, Eng JV, Sodahlon YK, Ter Kuile FO, Hawley WA (2010). Impact of mass distribution of free long-lasting insecticidal nets on childhood malaria morbidity: the Togo national integrated child health campaign. Malar J.

